# “Cut and Paste” Processes in the Search of Bioactive Products: One-Pot, Metal-free *O*-Radical Scission-Oxidation-Addition of *C*, *N* or *P*-Nucleophiles

**DOI:** 10.3389/fchem.2022.884124

**Published:** 2022-05-18

**Authors:** Marina Porras, Dácil Hernández, Concepción C. González, Alicia Boto

**Affiliations:** Instituto de Productos Naturales y Agrobiología del CSIC (IPNA-CSIC), Tenerife, Spain

**Keywords:** hypervalent iodine, O-radicals, one-pot processes, metal-free, radical-ion crossover, peptides, nucleosides, alkaloids

## Abstract

Hypervalent iodine reagents have been applied in many metal-free, efficient synthesis of natural products and other bioactive compounds. In particular, treatment of alcohols, acetals and acids with hypervalent iodine reagents and iodine results in *O*-radicals that can undergo a β-scission reaction. Under these oxidative conditions, derivatives of amino acids, peptides or carbohydrates are converted into cationic intermediates, which can subsequently undergo inter- or intramolecular addition of nucleophiles. Most reported papers describe the addition of oxygen nucleophiles, but this review is focused on the addition of carbon, nitrogen and phosphorous nucleophiles. The resulting products (nucleoside and alkaloid analogs, unnatural amino acids, site-selectively modified peptides) are valuable intermediates or analogs of bioactive compounds.

## Introduction

Hypervalent iodine reagents have proven very useful for the synthesis of natural products and other bioactive compounds (Dohi and Kita, 2016; Wang, 2021). A variety of methodologies have been developed, in many cases combining the hypervalent iodine reagents with other compounds, such as iodine, organic peroxides, TEMPO, and organic photosensitizers or metal catalysts in catalytic photoredox processes (Singh and Wirth, 2021; Le Du et al., 2021; Zhdankin, 2020; Chen et al., 2020; Wang and Studer, 2017; Yoshimura and Zhdankin, 2016; Wang and Liu, 2016; Zhdankin and Stang, 2008; 2002).

The use of metal-free procedures is particularly important for the synthesis of bioactive products, to avoid undesired contamination of the product, especially when large-scale synthesis is needed for bioactivity assays or industrial production (Dohi and Kita, 2016). Among these metal-free methodologies, the combination of hypervalent iodine reagents and iodine (Suárez reaction) is quite interesting for its operational simplicity, low reagent toxicity, reagent degradation during aqueous work-up, and easy product purification. This method has been used to generate *O*- and *N*-radicals that can undergo different reactions depending on the substrate and reaction conditions, mainly hydrogen abstraction and scission of the C_α_,C_β_-bond (β-fragmentation) (Stella, 2001; Suárez, 2001; Li et al., 2010; Wang, 2021).

Our group and others have described different applications of the β-scission of *O*-radicals, generated from alcohols, acetals or acids under Suárez conditions, to the preparation of bioactive products or their analogs (Saavedra et al., 2020; Cuevas et al., 2021 and references cited therein). However, in these protocols a further development was introduced: coupling these oxidative scission processes to the addition of carbon, nitrogen, phosphorous, oxygen, hydrogen, sulfur and other nucleophiles (Boto, Gallardo et al, 2006; 2007a; Boto, Hernández et al, 2008a,b; 2009; Batchu et al, 2014; Carro et al, 2017; Saavedra et al., 2018; Santana and González, 2020). In effect, the scission generates a *C*-radical that can be readily oxidized to a cationic intermediate when the latter is stabilized by adjacent groups. This is the case for tertiary *C*-radicals, or those adjacent to nitrogen or electron-rich oxygen functionalities (Boto et al., 1999–2004; 2005a; 2007a; 2007b; 2007c; 2007d; Francisco et al., 2001; Romero-Estudillo, 2013, 2015a, 2015b; Kiyokawa et al., 2017; André-Joyaux et al., 2019). When the substrates are aminoacids or β-hydroxyamines, an intermediate iminium ion is formed, while carbohydrate substrates afford oxycarbenium ion intermediates. These cationic species may then undergo nucleophilic addition; most reports describe the addition of oxygen nucleophiles (Chai et al., 1998a; 2005; Francisco et al., 2001; Boto et al., 2005a; 2007a; 2008a; Miguélez et al., 2012; Kiyokawa et al., 2018), but this minireview will focus on carbon, nitrogen and phosphorous nucleophiles. Since several transformations are carried out consecutively, with no need to purify the intermediates, these one-pot radical-polar crossover reactions save time, materials and energy with respect to the original conditions.

A selection of these one-pot scission-oxidation-addition of C, N and P nucleophiles methodologies is presented in this review, as well as the known or potential bioactivities of the products thus obtained.

## Oxidative *O*-RADICAL SCISSION-ADDITION of Carbon Nucleophiles

**FIGURE 1:**
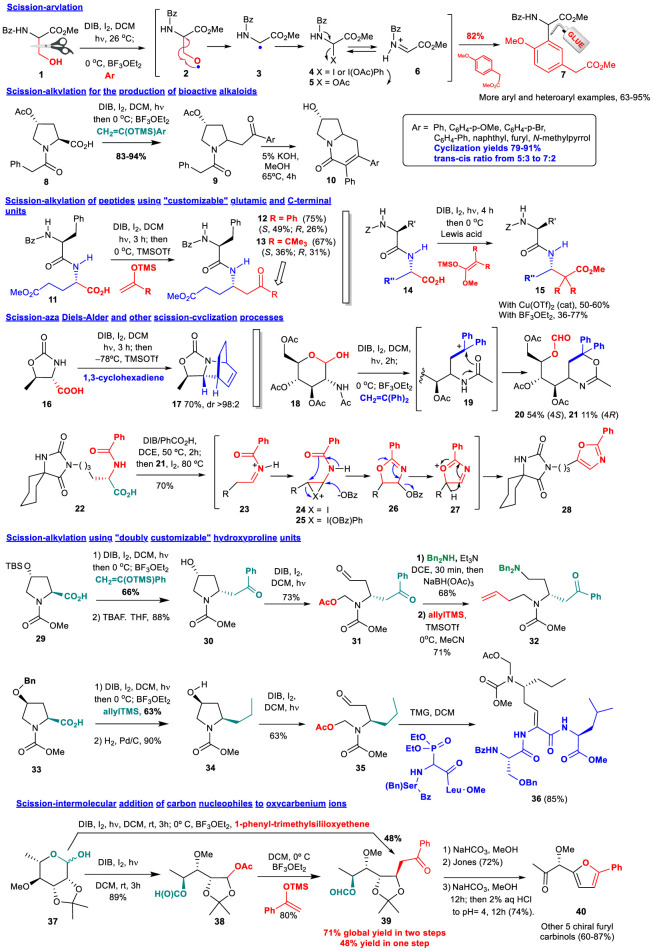
Scission-addition of *C*-nucleophiles.

One of the first applications of this process was the generation of aryl glycines, some of which displayed cytotoxic properties (Boto, Gallardo et al, 2007a; 2006). As shown in conversion 1→ 6 (Figure 1), treatment of serine derivatives 1 with hypervalent iodine reagents (such as diacetoxyiodobenzene, DIB) and iodine, under irradiation with visible light, generated an *O*-radical 2 that underwent β-scission to give a *C*-radical 3. The latter reacted with iodine or with the hypervalent iodine reagent to give an unstable intermediate 4 that was transformed into an α-acetoxyglycine 5. On treatment with a Lewis acid an acyliminium ion 6 was generated, that reacted with electron-rich arenes, to afford arylglycines such as 7 in good to excellent yields. Arylglycines are components of antibiotics such as nocardicins and vancomycin, anti-neurodegenerative agents and alkaloids (Williams and Hendrix, 1992; Boto, Gallardo et al, 2007a).

A related scission-alkylation reaction was used to prepare analogues of cytotoxic indolizidine alkaloids (Miguélez et al., 2013a). Thus, proline amides 8 underwent decarboxylation and the addition of silyl enol ethers derived from aryl methyl ketones, to give the substituted pyrrolidines 9. Cyclization under basic conditions afforded the two isomers of the desired bicyclic systems 10, which were separated and tested. Some of these alkaloid analogues displayed a promising activity, but interestingly, some monocyclic derivatives 9 also did, which could be useful to determine SAR relationships.

A variation of the previous scission-alkylation methodology was applied to the site-selective modification of peptides using “customizable” glutamic acid (Saavedra et al., 2012c) or C-terminal residues (Saavedra et al., 2009). In both cases, an oxidative radical decarboxylation took place, followed in the first case (conversion 11→ 12/13) by the addition of silyl enol ethers to give derivatives 12 or 13 in good global yields. In that way, an ordinary α,α-unit was converted into α,γ-peptide hybrids, which have elicited interest for their antimicrobial, antitumour, antihypertensive, and anti-Alzheimer properties, as well as their superior resistance to protease degradation (Ordóñez and Cativiela, 2007; Hernández et al., 2017).

In the second example, the substrate 14 was decarboxylated and subjected to the addition of silyl ketenes, to give substituted α,ß-peptide hybrids such as compound 15. These hybrids had unusual conformations, which could be used for drug or catalyst design (Saavedra et al., 2009; 2012a; 2012b). In addition, many α,β-hybrids have displayed promising activities such as the antitumour dipeptide bestatin (Ubenimex). Moreover, they are more resistant to degradation by peptidases, as evidenced with a series of α,β-peptide bradykinin cleavage inhibitors, whose half-life was greatly increased with respect to the α,α-analogues (Bauvois and Dauzonne, 2006; Aguilar et al., 2007).

The scission reaction can also be followed by a cycloaddition reaction. Thus, when substrate 16 was decarboxylated and treated with different dienes, it provided cycloaddition products such as 17 in good yields and excellent stereoselectivities (Boto and Romero-Estudillo, 2011). Other addition-cyclization reactions have been also described, such as the transformation of the α,ß-amino sugar 18 into the oxazines 20 and 21, through intramolecular cyclization of cationic intermediate 19 (Boto, Gallardo and Alvarez, 2012). In another example, the one-pot conversion of an amino acid into an oxazol (transformation 22→ 28), the reagent (diacetoxyiodo)benzene was transformed *in situ* into other (diacyliodo)benzenes; then, the substrate 22 was added and underwent an oxidative radical scission to give an acyliminium intermediate 23. This ion isomerized to an enamide, which reacted with iodine (or the hypervalent iodine reagent) to afford an intermediate 24, which experienced an intramolecular cyclization with opening of the halonium ring. The halogen group was then replaced by the acyloxy moiety (benzoate in the example) to give the oxazolidine 26. However, the reaction went on, with extrusion of the benzoate, formation of a cationic intermediate 27 and aromatization, providing the oxazole 28 in 70% global yield (Romero-Estudillo et al., 2014). These heterocycles can be found in many bioactive peptides, and are considered privileged structures (Boto, González et al, 2021).

Two scission reactions can be carried out in “doubly customizable” hydroxyproline units (Hernández et al., 2021). Thus, using the hydroxyproline substrate 29, a decarboxylation-alkylation was carried out, to give a 2-substituted pyrrolidine in good yield and excellent 2,4-cis stereoselectivity. After deprotection of the 4-hydroxy group, the resultant pryrrolidine 30 underwent a second *O*-radical scission, to afford compound 31, which presented two new chains which could be manipulated independently (conversion 31→ 32). Thus, the α-chain was subjected to a reductive amination, while the addition of allylTMS to the *N,O*-acetal gave an olefinic chain, that could be further diversified using olefin metathesis (Saavedra et al., 2019; Hernández et al., 2021).

In a second example (conversion 33→ 23), the decarboxylation-allylation of compound 33, followed by hydrogenation, afforded a 2-substituted pyrrolidine 34. The stereogenic center at C-4 determined, as before, the configuration at C-2, and thus, compounds 30 and 34 had opposite C2-stereochemistry. This result was translated to the second scission product 35, which was used in a Horner-Wadsworth-Emmons reaction to afford the peptide 36. This peptide has a dehydroaminoacid unit, which is often used in peptides to provide rigidity and a better interaction with biological targets. Dehydropeptides have displayed antimicrobial, antitumour and phytotoxic activities (Jiang et al., 2015; Siodlak, 2015).

The addition of *C*-nucleophiles to oxycarbenium ions derived from the scission of carbohydrates has also been studied, as shown by the conversion 37→ 39 (Boto, Hernández et al, 2007c). In this case, however, the one-pot procedure was less efficient than the two-step process, where the intermediate acetate 38 was isolated and then treated with a Lewis acid and the *C*-nucleophile. Other examples were also studied, with similar results. The resultant products were converted in a few steps and with high optical purity into chiral furyl carbinols, which are useful precursors of bioactive products, such as the selective antifungal populacandin D (Balachari and O’Doherty, 2000), or KDO, a vital component of the Gram-negative bacteria cell wall (Martin and Zinke, 1991).

## Oxidative *O*-Radical Scission-Addition Of *N*- and *P*-Nucleophiles

**FIGURE 2:**
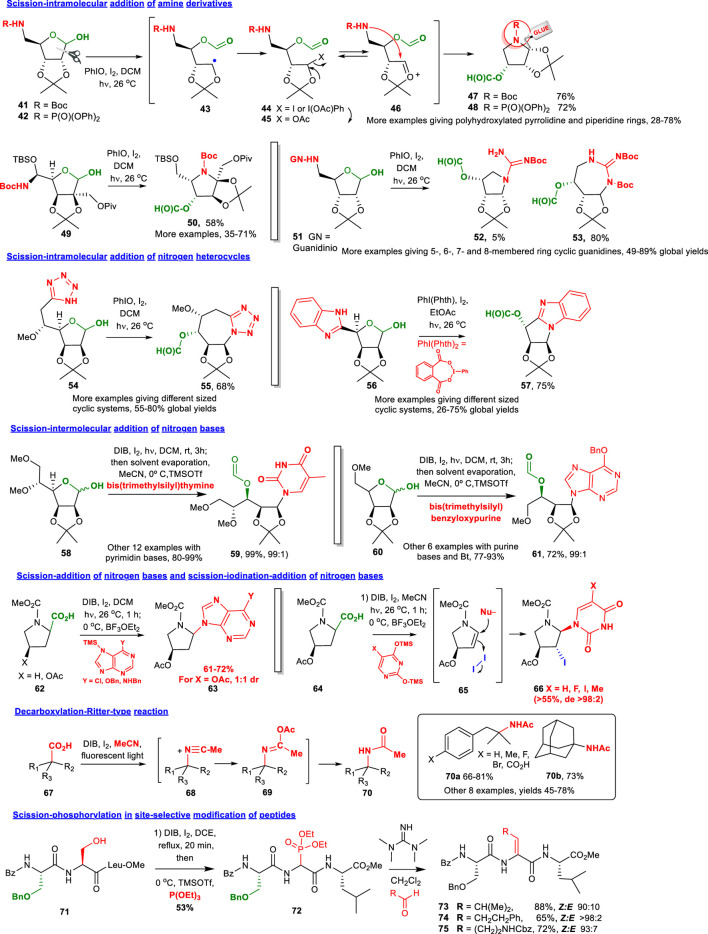
Scission-addition of *N*- and *P*-nucleophiles.

The scission-addition of nitrogen nucleophiles provides other families of potentially bioactive products, such as iminosugars and nucleoside analogues (Figure 2). Many iminosugars have displayed a potent activity as glycosidase inhibitors, while many nucleoside analogues have been used as antimicrobial or antitumour agents (Horne et al., 2011; Drug Bank, 2021). Given the potential in this field, the main goal of our research has been focused on synthesizing new classes of iminosugar derivatives starting from carbohydrates.

As shown in the conversion 41/42→ 47/48 (Figure 2), the cleavage of the carbohydrate C1-C2 bond forms a C-radical 43 that reacts with iodine or the hypervalent iodine reagent to afford a halogenated intermediate 44 (Francisco et al., 2001). Indeed, some iododerivatives (X = I) are stable enough to be isolated and characterized (Francisco et al., 1998b). But in many cases, the intermediate is rapidly converted into an acetoxy compound (*eg* product 45). This acetoxyderivative 45 is in equilibrium with its oxycarbenium ion 46, which can undergo the intramolecular addition of nitrogen nucleophiles, to give nitrogen heterocycles such as 47 and 48. A related conversion of aldoses to ketoses 49→ 50 also took place in satisfactory yield (Santana et al., 2013). This methodology, which represents an efficient alternative to the Lobry de Bruyn-Alberda van Ekestein alkaline isomerization, used readily available aldoses as starting material. Despite the steric hindrance to nucleophile approach, the reaction proceeded smoothly, affording ketoses such as 50, where a new quaternary center with a desired configuration has been incorporated.

Following this strategy, a variety of novel polyhydroxylated heterocyclic compounds have been recently prepared. For example, cyclic guanidines derived from carbohydrates were synthesized as shown in transformation 51→ 52/53 (Santana and González, 2020). The bidentate nucleophilic character of the guanidinium group opened the possibility to differentiate between the two non-equivalent nitrogen atoms, which made the proposed methodology more versatile, as endocyclic or exocyclic guanidines of different sizes (5-, 6-, 7- or 8-membered rings) could be generated in good yields (85% in the example shown). The guanidinium moiety appears in natural products with potent biological activities, such as saxitoxin, tetrodotoxin or crambescin, and also drugs such as antiplasmodium compounds (Alonso-Moreno et al., 2014; Perry et al., 2019).

This versatile strategy has also provided a battery of sugar tetrazoles and benzimidazoles (Paz et al., 2012; André-Joyaux et al., 2019). Remarkably, in the key cyclization step these aromatic nitrogen heterocycles were used for the first time as nucleophiles. The method efficiently afforded polycyclic systems such as 55 and 57 in good yields. Tetrazole and benzimidazole are privileged structures, and tetrazoles are found in many fungicides. These heterocycles have been used to protect crops since the 1970s, due to their low or moderate toxicity, broad fungicide spectrum and potent systemic action (Pernak et al., 2015). Benzimidazoles have displayed potent antimicrobial properties, and carbohydrate-bound benzoimidazoles (pseudonucleosides) are antiviral agents (Verma et al., 2016).

Nitrogen bases can also add to oxycarbenium ions, as shown in the conversions 58→ 59 and 60→ 61 (Boto, Hernández et al, 2007b). Both purine and pyrimidine bases reacted in good to excellent yields. The acyclic nucleosides have elicited much interest as antivirals (de Clercq, 2005). In addition, the reaction of nitrogen bases with acyliminium ions, as in transformation 62→ 63 (Boto, Hernández and Hernández, 2010a), afforded azanucleosides, a class of compounds with antimicrobial, anticancer and enzyme inhibitor properties (Hernández and Boto, 2014). Interestingly, when the scission reaction was carried out in acetonitrile instead of the more usual, less polar solvent dichloromethane (conversions 64→ 66), the initial iminium ion isomerized to an enamine derivative such as 65, which reacted with iodine and then with the nucleophile to give the final iodoazanucleosides (eg compound 66; Boto, Hernández and Hernández, 2010b).

The decarboxylation of acids to give tertiary cationic species which reacted with the solvent (acetonitrile) to afford Ritter-type products (conversion 67→ 70), was studied by Kiyokawa et al. (2017) giving hindered amines such as 70a and 70b in good yields. A decarboxylative amination where boron Lewis catalysts were used instead of iodine was reported by Narobe, König et al. (2022).

Finally, the one-pot scission-addition of phosphorous nucleophiles was studied for the site-selective modification of peptides (Boto, Gallardo et al, 2005b; Saavedra et al, 2012d; 2018). After the oxidative radical scission a phosphite was added, and an aminophosphonate such as compound 72 was formed. A Horner-Wadsworth-Emmons reaction with different aldehydes was carried out to give a peptide with a dehydroaminoacid unit, which increased the system rigidity. The reaction took place in good yields and a high *Z* stereoselectivity, even when interior positions were functionalized. The introduction of dehydroaminoacids into peptides can improve their interaction with their biological targets and their resistance to proteases. Therefore, dehydroaminoacids are components in a variety of bioactive natural peptides and drugs (Jiang et al., 2015; Siodlak, 2015).

The tandem decarboxylation-phosphorylation process was recently adapted by Viveros-Ceballos et al. (2021) to prepare tetrahydroisoquinoline-3-phosphonic acids, which are key components of enzyme inhibitors and other bioactive products. In another example, nucleotide analogs were formed by a decarboxylation-phosphorylation reaction (Miguélez et al., 2013b). Although the authors do not report the bioactivity for this set of compounds, clearly this methodology would be valuable for the preparation of chemical libraries for structure-activity relationships.

## Conclusion and Outlook

The use of metal-free methodologies for the synthesis of bioactive products is a hot area in pharmaceutical chemistry, and hypervalent iodine reagents have proven very useful to achieve this goal. Moreover, among metal-free synthetic methodologies, particularly interesting are those which carry out the transformation of readily available natural products into added-value bioactive compounds using one-pot ‘cut and paste’ processes.

This mireview focuses on the methods which use the generation and scission of *O*-radicals in their key step, followed by the addition of carbon, nitrogen and phosphorous nucleophiles. These methodologies include scission-alkylation, scission-arylation, fragmentation-Diels Alder and other inter- and intramolecular cyclization processes, scission-Ritter, fragmentation-addition of nucleobases, and scission-phosphorylation. A range of products can be obtained from simple substrates such as organic acid and alcohols, amino acids, carbohydrates and peptides. Among those interesting for their potential bioactivity are alkaloid and nucleoside analogues, heterocycles, aminophosphonates and other amino acid analogues, and site-selective modified peptides and peptide hybrids.

In most cases, these strategies afforded high added-value products in good to excellent yields, operational simplicity and easy work-up and product purification. These processes offer a quick route to families of many bioactive products (such as glycosidase inhibitors and other antimicrobial or cytotoxic compounds) and also to some new compounds containing privileged structures, whose biological properties deserve to be further studied. This could be a goal for the next future in this area.

The work carried out up to now highlights the opportunities offered by these sustainable metal-free, one-pot methodologies, where many other substrates and nucleophiles remain to be explored. Since structural diversity often translates into biological diversity, future efforts in the topic could provide new promising bioactive compounds and drug candidates.
